# 

**Published:** 1886-12

**Authors:** 


					﻿A/hoie NumbePry^j
DECEMBER, 1886.
Vol. LI 11. Number 6
THE
CHICAGO RIEOrarJOURNAL AND EXAMINER
EDITORS:
S. J. JONES, M.D., LL.D.
HAROLD N. MOYER, M.D
PUBLISHED MONTHLY BY
TELE CLARK & LONGLEY OOLZEELA-LTOC,
Under direction of
THE CHICAGO MEDICAL PRESS ASSOCIATION,
308-316 Dearborn Street.
				

